# Utilization obstacles to hypertension services provided at comprehensive health centers: a content analysis study

**DOI:** 10.1186/s12961-023-00984-w

**Published:** 2023-05-26

**Authors:** Farzad Faraji-Khiavi, Sasan Ghorbani Kalkhajeh, Behnam Gholizadeh, Behnaz Dindamal

**Affiliations:** 1 Atherosclerosis Research Center, Ahvaz Jundishapur University of Medical Sciences, Ahvaz, Iran; 2 Healthcare Services Management, Department of Public Health, School of Health, Abadan University of Medical Sciences, Abadan, Iran; 3 Department of Cardiac Surgery, Atherosclerosis Research Center, Ahvaz Jundishapur University of Medical Sciences, Ahvaz, Iran; 4 Department of Health Services Management, School of Health, Ahvaz Jundishapur University of Medical Sciences, Ahvaz, Iran

**Keywords:** Utilization, Hypertension, Comprehensive health centers, Content analysis

## Abstract

**Introduction:**

Hypertensive patients' failure to refer to health centers for the management of their disease is the most fundamental public health challenge in most societies. The aim of this study was to identify the utilization obstacles to hypertension services provided at comprehensive health centers (CHCs) from the perspective of patients and the staff of health centers.

**Methods:**

This was a qualitative study using conventional content analysis which was conducted in 2022. Participants included 15 hypertensive patients referring to CHCs and 10 staff (including personnel of CHCs and expert staff) of Ahvaz Jundishapur University of Medical Sciences, Ahvaz, southwest of Iran. Data were collected using semi-structured interviews. Interviews were analyzed using content analysis method, and coding was done manually.

**Results:**

In total, 15 codes and 8 categories were extracted from the interviews which were organized in two main themes of "individual problems" and "systemic problems". More particularly, the main theme of “individual problems” involved attitudinal obstacles, occupational obstacles, and economic obstacles. The main theme of "systemic problems" included educational obstacles, motivational obstacles, procedural obstacles, structural obstacles, and managerial obstacles.

**Conclusion:**

In order to address individual problems associated with the patients' failure to refer to CHCs, we need to take appropriate measures. These include using motivational interviewing techniques and utilizing the effective activity of healthcare liaisons and volunteers in CHCs to increase patients' awareness and change their negative attitudes and misconceptions. To solve systemic problems, it is imperative that effective training courses be held for health center staff.

## Introduction

Hypertension is a well-known medical condition which is the most important controllable risk factor for cardiovascular diseases, stroke, and chronic kidney disease [1]. Many factors contribute to the presentation and continuation of hypertension. Genetically, many single nucleotide polymorphisms (SNPs), genes, and epigenetic factors are associated with hypertension. Thus, people with these hereditary factors may have a genetic predisposition to develop hypertension [2]. Environmental factors such as obesity, alcohol consumption, sedentary lifestyle, and high sodium intake also contribute to the development of hypertension [3].

The prevalence of hypertension is increasing worldwide due to an aging population, and about 1.28 billion adults aged 30–79 worldwide have been reported to suffer from hypertension [4]. According to the WHO’s non-communicable diseases (NCD) country profile (2018), the prevalence of hypertension among Iranian adults aged 18 + was 17% [5]. Meanwhile, in a 2019 study, the overall prevalence of hypertension among Iranian adults was 48%, according to the new ACC guideline [6]. In 2015, about 8.5 million deaths were caused by SBP > 115 mmHg. [7] Hypertension imposes a high economic burden on society, and is accompanied with a high incidence of debilitating complications [8].

Hypertension control includes identification of people at risk, appropriate treatment of hypertensive patients through medication or lifestyle changes, and follow-up to ensure that patients adhere to treatment and that hypertension is controlled [9]. The implementation of these recommendations in practice is less than optimal, and the hypertension control process may fail due to the inability to overcome obstacles related to the patient, the health care provider, or the health system. Thus, reduced treatment acceptance by the patient affects the follow-up care and the desire of patients to go to health centers [10]. In various studies, the following have been reported as obstacles to receiving services and follow-up care: transportation [11], conflicting working hours with service delivery hours [12], lack of easy access to comprehensive health centers (CHCs) [13], short consultation time with service providers in health centers [14], not receiving adequate nutritional recommendations by nutritionists in CHCs [15], follow-up care only for minor problems due to high costs [16], poor provider-patient communication [17], and patients' lack of trust in the services provided [18].

CHCs are at the front line of the prevention of non-communicable diseases. They must be prepared to control hypertension, as a major health risk factor, in all covered populations and carry out the initial stages of treatment and follow-up. Identifying utilization obstacles is a major step in developing and implementing actions and policies aimed at removing these obstacles and thus achieving effective treatment for these patients. In this way, by extending the coverage of care by health care providers, individuals suffering from or susceptible to hypertension can be detected on time and put on the path of treatment. These obstacles can generally lead to insufficient knowledge of patients about their disease process, a negative impact on their self-care process, dangerous progression of the disease, dissatisfaction of personnel, and poor performance of CHCs in providing high blood pressure services and controlling this medical condition in an appropriate and timely manner.

The literature lacks sufficient studies exclusively identifying the utilization obstacles to hypertension services provided at CHCs, and most of the studies have generally addressed the "obstacles to care" that lead to the low utilization of patients from CHC services. On the other hand, the few studies that have investigated the utilization obstacles to services provided at CHCs did not focus solely on hypertensive patients, and their target groups included other groups of patients in addition to hypertensive patients. In most of these studies, the utilization obstacles to services were investigated only from the patient’s point of view, while in the present study, the opinions of the staff were also scrutinized. Therefore, the present study specifically identified the utilization obstacles to hypertension services provided at CHCs in Ahvaz, southwest of Iran from the perspective of both the patients and the staff.

## Methods

This was a qualitative study conducted in 2022 using a conventional content analysis approach. Qualitative content analysis is a method used for subjective interpretation of qualitative data through systematic classification process of coding and identifying themes [19]. The aim of this study was to identify the obstacles to hypertensive patients' utilization of CHC services at Ahvaz, Iran, for the control and care of their disease.

### Study setting

Ahvaz is a metropolis in the southwest Iran. It has 46 CHCs that are affiliated to universities of medical sciences and provide primary health care services (such as family health, health education, nutrition, mental health, etc.). These CHCs play a significant role in facilitating the access of people to primary care and maintaining and improving health. Participants were selected from 10 CHCs located in Ahvaz.

### Participants and sampling

The participants of this study included hypertensive people who referred to CHCs of Ahvaz and healthcare staff (including the personnel of these centers and the expert staff of Ahvaz Jundishapur University of Medical Sciences). Overall, 25 people participated in the research, of whom 15 were patients and 10 were staff (7 personnel of CHCs and 3 staff experts of university). The inclusion criteria for patients was as follows: having hypertension (SBP ≥ 130 mmHg and DBP ≥ 80 mmHg), aged 30–60 years, being diagnosed with hypertension at least one year prior to commencement of the study, and taking antihypertensive medication. We limited our study to patients aged 30–60 years because the prevalence of hypertension before the age of 30 years is relatively low, and care goals at older ages receive more priority and importance. On the other hand, in Iran, CHCs follow another protocol for patients over 60 years of age. Patients were excluded from the study if they had serious physical or mental disabilities, or suffered from anxiety, depression, or other psychological problems. Inclusion criteria for the staff were: having at least one year of work experience in the centers and being active in the field of work related to the hypertension control and care program.

The participants were selected based on purposive sampling method. In this method, the researcher decides what needs to be known and sets out to find people who can and are willing to provide the information by virtue of knowledge or experience [20]. Some of the participating patients were among the patients who visited the clinic where one of the researchers (a cardiologist) worked, but the sampling was not limited to them. In this study, none of the participants wanted to withdraw from the study, and all the selected individuals participated in the interviews.

### Data collection

Individual face-to-face semi-structured interviews were conducted to collect the data. The place and time of the interview was set at the participants' convenience, and after finalizing the interview time with those who met the inclusion criteria, the researchers started conducting the interviews. Two researchers (FF and SGh) who had notable experience in interviewing and conducting qualitative research were selected as interviewers. The interviewers were not physicians, nor was there any patient-physician relationship between them and the participants. The interviews included only the interviewer and the interviewee and nobody else.

According to the interview guide, simple and general questions were asked at the beginning, and according to the answers and the experiences of the participants, the interview continued towards more specific and detailed questions. After designing the interview guidelines, their validity were confirmed by eight faculty members and CHCs experts. The patients were asked key questions such as: "What factors prevent you from visiting your designated CHC?", "Are there other reasons related to the health system and the CHC that have caused you not be visiting regularly? Please explain to me" and "What conditions do you think should be provided to encourage you to visit CHC regularly to control your disease?" Experts were also asked: "Why do you think patients with hypertension do not refer to CHCs for the care and control of their disease?" and "What obstacles do you think patients face that are unique to CHCs?".

Each interview lasted from 30 to 45 min. Prior to the interview, the purpose of the study was explained to the participants. The interviews were recorded using a voice recorder with the participants’ permission and consent. Field notes were also taken during the interviews by interviewers. In the next step, the interviews were transcribed verbatim. The transcriptions of the interviews were provided to the participants to confirm their accuracy and to give feedback. Discrepancies were resolved, and parts that did not express their views were corrected. All interviews took about four months to finish. There was no need to repeat the interview for any of the participants. Interviews continued until data saturation.

### Data analysis

After the interviews were conducted, they were transcribed verbatim. Interviews were analyzed using content analysis method, and data coding and categorizing were done manually.

According to the content analysis method, after conducting the interviews, the researchers tried to get acquainted with the data well by carefully and repeatedly reading the transcript of the interviews in the first step. In the second step, important sentences and words were extracted from the transcription of the interviews. The third step involved conceptualizing and coding the extracted semantic units. Data coding was done by two coders separately, and then the codes were compared, and discrepancies were resolved. The comparison of initial codes and consensus on the final codes were done by all researchers of the study. In the fourth step, after re-reading the codes for several times, concepts were formulated and placed in subject clusters, and thereby the main themes emerged. In the final stage, the obtained structure was approved by the patients and the staff.

### Scientific trustworthiness of the results

In order to verify data validity, Lincoln and Guba's four criteria were followed: credibility, confirmability, dependability, and transferability [21]. To enhance the credibility of the findings, sampling continued until data saturation. Also, the results of the interviews were provided to the participants for confirmation. In other words, the review of data and results done by the participants helped to identify and remove the researchers' biases. Another way to increase the credibility of the data is to ensure that the data are appropriately covered by themes and sub-themes. In this way, irrelevant data are removed, and relevant data are included. To increase the dependability of the research results, we used external check. Confirmability of findings was enhanced via investigator triangulation which involves engaging more than one researcher in gathering, analyzing, and interpreting the data. The aim of investigator triangulation is to make the bias that may occur due to a single researcher’s bias less likely. The use of teamwork (investigator triangulation) reduced personal taste and controlled researchers' bias. In addition, in order to reduce this bias, the opinions of expert staff in university were used to select relevant sites and personnel with high knowledge. Transferability of data was ensured by offering a comprehensive description of the subject, participants, and data collection and analysis procedures.

### Ethical considerations

Prior to the interview sessions, the following ethical considerations were sought: obtaining verbal consent from the participants, ensuring the confidentiality of information and names, giving the participants the right to withdraw from the research at any time, and informing them of the objectives of the study. Also, after obtaining approval from the Ethics Committee of Ahvaz Jundishapur University of Medical Sciences (Ref. ID: IR.AJUMS.REC.1401.003), the researchers presented a letter of introduction to the research sites (i.e., CHCs of Ahvaz city) and conducted the interviews. All methods were carried out in accordance with the relevant guidelines and regulations.

## Results

There were 25 interviewees in this study (15 patients and 10 staff). The majority of the interviewees were female, and most of them were in the age range of 45–60 years and had a bachelor's degree. The married participants outnumbered their single counterparts. Table 1 shows the demographic characteristics of the participants.

**Table 1:** Demographic characteristics of the interviewees

Demographic profile	Category	Number (%)
Gender	Female	15 (60)
	Male	10 (40)
Age	< 30	2 (8)
	30–45	11 (44)
	46–60	12 (48)
	> 60	0 (0)
Educational attainment	< Associate degree	9 (36)
	Bachelor’s degree	10 (40)
	> Master’s degree	6 (24)
Marital status	Single	7 (28)
	Married	18 (72)
	Total	25 (100)

After analyzing the obtained data, the main codes, categories and themes (problems), which were associated with utilization obstacles to hypertension services provided at CHCs, were identified. In total, 15 codes and 8 categories were extracted from the interviews of which 5 codes and 3 categories were related to the main theme of "individual problems" and 10 codes and 5 categories were related to the main theme of "systemic problems". Table 2 lists the semantic units regarding individual problems associated with hypertensive patients' utilization of services at CHCs along with the categories and codes identified.

**Table 2:** Reasons of hypertensive patients' failure to utilize CHCs’ services from the perspective of the staff of health centers and the patients (individual problems)

Main theme	Category	Code	Semantic units
Individual problems	Attitudinal obstacles	Not receiving the expected results	• Not receiving the expected results in the short term due to the chronic course of the disease • Not receiving the expected results in the short term due to long-term treatment of the disease
		Cultural flaws of the target population and low sensitivity to the disease	• Underestimation of the disease by patients' families • Some patients’ ignorance of the disease and not being worried about its progression • Ignoring the follow-up care and refusing to go to the center due to fear of getting COVID-19
		Misconception	• Some patients' availing themselves of self-medication • The misconception of male patients that CHCs are only for women and children • Clients' misconception that the purpose of referral and receiving care is only to receive the pills for free
	Occupational obstacles	Busy schedule of patients	• Highly busy schedule of patients • Prioritizing work over care due to official working hours
	Economic obstacles	Low income of patients	• Inability to afford the commuting expenses for referring to CHCs • Inability to afford the possible care costs

Some of the staff pointed to the frivolousness of in-service training as a factor contributing to patient dissatisfaction and thus the patients' failure to go to CHCs. "Sometimes our training sessions are held by staff who are at our level or even lower than us, and the sessions are of little quality. Obviously, not much change is created to improve patient care" (Staff 3). A number of health care staff said that one of the reasons for patients' failure to refer to the CHCs was their lack of knowledge about the secondary problems of the disease. "Some patients with hypertension do not know about the other problems the disease may bring about in future" (Staff 6). The long-term nature of the treatment of the disease was mentioned as a preventive factor which made hypertensive patients not refer to CHCs. "Treating my hypertension is very time-consuming and that’s why I think I’ve lost my motivation to go to the center and pursue my treatment" (Patient 7). Many patients cited the fear of contracting COVID-19 as a factor in ignoring their current illness and therefore not seeking care. "I've come to the center much less often since COVID-19 showed up. I can control my hypertension at home, but the environment in the centers is very infectious these days. If I get infected, something worse may happen" (Patient 4). According to a number of staff members, the patients' self-medication had to some extent led to their low utilization of services at CHCs. "Some of our patients do not believe in the services of the center and their avid interest in self-medication has prevented them from coming to the center" (Staff 8). Work issues were raised by patients as one of the decisive utilization obstacles to hypertension services provided at CHCs. "I am a government employee and I can’t go to the center because of my work time limits, because the centers are closed after office hours" (Patient 9). Many patients cited financial issues and high costs as one of the main reasons for not going to CHCs. "I don’t go to the center because I have to pay a lot for getting a taxi, which I can’t afford" (Patient 2).

Table 3 shows the systemic problems associated with hypertensive patients' utilization of services at CHCs in Ahvaz.

**Table 3:** Reasons of hypertensive patients' failure to utilize CHCs’ services from the perspective of health staff and the patients (systemic problems)

Main theme	Category	Code	Semantic units
Systemic problems	Educational obstacles	Lack of comprehensive and efficient training for employees	• No training for the newly recruited personnel who join the health system • Frivolousness of in-service training and retraining • Inefficient training sessions with high quantity and low quality • Insufficient attention to the use of new teaching methods
		Insufficient awareness about the disease	• Unawareness and inadequate knowledge of patients' family members about the disease • Lack of the necessary knowledge of some patients and their family members about secondary problems of the disease
	Motivational obstacles	Low staff motivation	• Lack of necessary and relevant incentives for executive staff
	Procedural obstacles	Multiple staff activities and lack of time to care for patients	• No sufficient patient time allocation due to busy staff • High burden of referrals to the center physician and thus ignoring patients
		Problems associated with information registration process	• Time-consuming registries and forms for recording information
		Heavy bureaucracy in the health system	• Long waiting lists
	Structural obstacles	Lack of sufficient amenities	• Disintegrated services and lack of facilities • Lack of space in CHCs and lack of adequate amenities and restrooms
		Lack of specialized staff	• Lack of knowledgeable and experienced manpower and some staff members’ urge to work in multiple centers • Lack of a planning officer in the urban CHCs
		Problems associated with access to CHCs	• The long distance to CHCs and the length of time taken to reach CHCs • The physical condition of people and the difficulty of traveling from home to CHCs • Location of CHCs
	Managerial obstacles	Problems associated with management of CHCs	• Presence of physicians without executive background in CHCs • Short-term and provisional presence of physicians in CHCs • CHCs managers' ignorance of management of hypertension • Therapeutic view of physicians toward health services • Poor insurance supervision over the treatment process of patients

The experts of the CHCs stated that their low motivation is an influential factor contributing to dissatisfaction of patients and their failure to refer to the center. "We haven’t received an incentive for a long time, and with this workload and a lot of exhaustion, our motivation has really reduced" (Staff 1). Some patients were dissatisfied with the high workload of the staff and therefore did not want to visit the CHCs regularly. "The staff at this center are very busy. Sometimes, they don't spend enough time on guiding me, and I’d rather not waste my time here" (Patient 3). The length of the data entry process was another factor that the patients stated would make them tired. "It takes a long time for a health care provider to enter the relevant information into their own system or forms. I don't have much time to stay in the center, and this waste of time makes me tired" (Patient 10). Another manifestation of the identified systemic problems was the long waiting queues. "I have to wait to receive services at the center, which makes me tired and not willing to visit the center the next time" (Patient 5). Many patients complained about the lack of adequate facilities, including amenities such as restrooms and the lack of specialists they needed. "I have to use the restrooms regularly, but the center's restrooms are not very clean" (Patient 8). "The specialists available here do not fit my needs. I need to get services from a hypertension specialist" (Patient 1). Obstacles to having access to the centers were among the reasons why patients with hypertension did not visit the CHCs. "There is no CHC near where I live and I have to travel long distances, so it's not possible for me to visit regularly" (Patient 6). Problems with managing the centers were also cited by most staff members. "The director of the center does not pay much attention to issues related to the follow-up of care for this disease. He simply does his job and then leaves. This has made us pay less attention to this issue" (Staff 10). "When there are officials in some medical centers who have no executive background and prioritize treatment over health, this attitude may be instilled into their staff as well" (Staff 4).

**Fig. 1:**
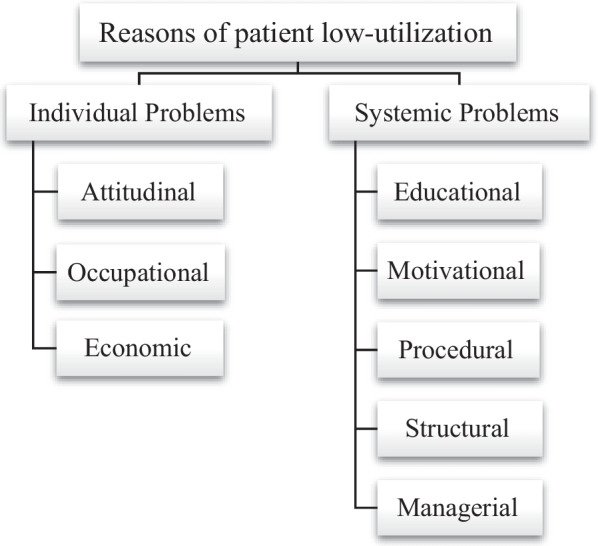
Reasons of hypertensive patients' low-utilization of CHC services from the perspective of staff and patients

Based on the information in the above tables, in general, the obstacles obtained from the interviews can be summarized as follows (Fig. 1). It should be noted that procedural and structural problems were obtained from interviewing patients while managerial and motivational problems were obtained from interviews with the staff of CHCs and expert staff.

## Discussion

In this study, the reasons why patients with hypertension refuse to refer to CHCs for care and control of their disease were elucidated by conducting interviews. Overall, the most obvious reasons for these patients' low utilization of services were a combination of individual and systemic problems, which will be discussed below.

### Individual-attitudinal problems

In the category of individual-attitudinal problems, some of the main reasons for hypertensive patients' low utilization of CHCs’ services include the following: not receiving the expected results in the short term due to the long course of treatment, having no fear of disease progression, and male hypertensive patients' misconception that CHCs are a place for women and children. The result of such attitudes is catastrophic: these patients tend to be reluctant about self-care, not adhere to treatment, and consequently refuse to go to the CHCs. All of these run counter to patient adherence to treatment which is one of the most significant measures to control hypertension [22]. In order to solve these problems, it is imperative to use appropriate methods to change the negative attitudes of patients and encourage them to change their behavior and adhere to their treatment. One of these methods is increasing patient awareness through appropriate and effective activities of health volunteers and liaisons in CHCs. A case in point is using motivational interviewing techniques, which are performed with the aim of stimulating the individual's intrinsic motivation to change behavior [23]. These techniques have been used in recent years to increase adherence to treatment, improve nutrition, and promote physical activity in people with diabetes [24], chronic obstructive pulmonary disease [25], and hypertension [26]. Ogedegbe et al. [26] admit the effectiveness of these techniques in promoting commitment to implementation of therapeutic interventions in patients with hypertension. Therefore, it seems that motivational interviewing can have positive effects on changing attitudes and behaviors, people's adherence to implementation of treatment interventions, referral to CHCs, and finally on hypertension control.

One of the problems in this category was the patients' neglect of follow-up care and failure to go to the centers out of the fear of getting COVID-19. With the rapid spread of COVID-19 across the world, countries’ potentials to address and respond to NCDs have been impacted. According to a WHO report, more than 50 percent of 122 studied countries experienced partial or complete disruptions to hypertension-related services based on a rapid assessment [27]. Policymakers of the health system should use appropriate strategies to ensure the capacity to provide, adapt and use resources in different shock and crisis scenarios. It is necessary and beneficial to use the experiences of different countries in increasing the resilience of the primary health care system, especially the care provided in CHCs. For example, the use of innovative methods in providing services, especially when a crisis occurs, leads to increased access of patients to health services [28].

### Individual-occupational problems

In this category, patients' busy schedules and their type of occupation and working hours were among the obstacles to utilizing CHC services. Due to the fact that some patients were employed in the morning shift and their working hours coincided with the time of providing health services at these centers, it was not possible for them to refer there and receive services, which is consistent with the results of Davoodi et al. [29]. In Papa et al.'s study on adults in urban and rural areas of Greece, the second reason why people did not refer to CHCs in order to receive health services was their busy schedule and lack of enough time [30]. Facilitating the conditions to provide services to this group of patients can help control hypertension as much as possible. For this purpose, various activities can be done. Performing health care practices actively at the workplace of patients or providing services in the afternoon are two options that could be taken into consideration.

### Individual-economic problems

The problems grouped under this category include: inadequate financial resources to continue care and pay for transportation costs. These economic obstacles were repeatedly raised by patients. According to the results of a collection of health studies in countries that are members of the Organization for Economic Development Cooperation, inadequate financial resources were the main reason of not referring to receive the required services among people whose income is below average [31]. Yee et al. found that women with financial difficulties often face obstacles to accessing health care, and some cited high costs that the patient has to pay as reasons for not referring to health care facilities [32]. In the present study, some patients could not pay for the health care services or cover the transportation costs due to economic problems, which is in line with the results of Davoodi et al. [29]. In this regard, it is expected that organizations that provide health services rely on public funding, as charities do, in order to expand their services.

### Systemic-educational problems

In the category of systemic-educational problems, one of the reasons why patients with hypertension refuse to refer to CHCs for the care and control of their disease was the lack of complete and efficient training of the staff. For example, one of the most important responsibilities of service providers is the proper measurement of blood pressure, and given the importance of this issue, it is necessary to optimize the performance of service providers in this regard. Padwal et al. showed that it is very important and sensitive to ensure personnel training, periodical retraining, and certification for accurate blood pressure measurement [33]. According to the findings of the present study, it is imperative to pay more attention to the quality and principles of staff training, and for this training to be effective, competent instructors should be employed. Another reason for the patients' failure to refer to CHCs is their lack of appropriate knowledge about the disease and its secondary problems. The effectiveness of any training program depends on the correct implementation of theories of behavioral sciences, and researchers have adopted different methods in order to bring about change in behavior. One of these methods that is effective in health education is the health belief model (HBM) according to which behavior is a function of the individual's knowledge and attitude. The results of a previous study showed that educational programs based on the HBM effectively promote preventive behaviors against hypertension among people [34]. Therefore, by considering the particular physical-mental conditions of patients, education and intervention based on this model can lead to enriching their awareness and thus their careful treatment behaviors.

### Systemic-motivational problems

In the category of systemic-motivational problems, one of the reasons patients with hypertension refused to visit CHCs was attributed to lack of incentives for the staff working at centers. It is a well-known fact that human resources are the main source of value creation in any organization, especially CHCs. Therefore, motivating them to increase productivity at work using various mechanisms such as rewards is one of the most vital duties of any organization [35]. In addition to its financial component that involves meeting the needs of low-income employees, the reward strategy includes a non-financial component which is associated with creating a sense of appreciation and job autonomy in employees and has a significant potential to change employee behavior [36]. A study by Waruni showed that giving intrinsic and extrinsic rewards leads to a sense of motivation in employees, and as a result leads to higher levels of performance [37]. Therefore, receiving organizational rewards can play a pivotal role in improving employee performance and has a positive effect on patient satisfaction and better utilization of services.

### Systemic-procedural problems

The chief systemic-procedural problems were: time constraints for treating patients, the time-consuming process of information registration, and the long waiting time due to the multiplicity of staff activities and the congestion of centers. The lengthy process of information registration due to the need to fill out many forms and the software problems, causes both congestion in the centers and dissatisfaction of people at the time of referral. Congestion in the centers has been mentioned as one of the main obstacles to providing timely services in a previous study [38]. Time is a crucial element when it comes to being responsive to clients and patients, and these are more satisfied with centers that arrange the time of follow-up and treatment sessions according to the patient's occupations, quickly admit the patient, define the time and duration of treatment for the patient, and take into account the patient's opinion about the number of weekly sessions, and the duration and time of counseling and treatment [39]. According to the results of previous studies, it can be stated that the Six sigma method can be used to reduce patient waiting time which is one of the factors causing dissatisfaction and to enhance the quality of services provided.

### Systemic-structural problems

The systemic-structural problems include cases such as lack of amenities, equipment, required specialties, and disintegration of services, which are in line with the results of Davoodi et al. [29] In a research conducted by Vitaleh to evaluate obstacles to health care services, local health staff stated that the increase in service provision and the services themselves can increase access to these services [40]. Effective provision of health services is seriously influenced by human resources, and there are major concerns about the inappropriate number of employees, as well as their type of expertise, distribution and performance [41]. Over the past half century, human resource planning in the health sector has received particular attention in order to distribute health workers in a way to achieve more equality in access to services, orientate educational planning, and employ staff to fulfill the goal of "health for all" through primary health care practices. On the other hand, the standard setting process for facilities can play a significant role in accelerating the process of equipping health care centers [42]. Other causes of patients’ non-utilization include the spatial distance between the clients' place of residence and the CHCs, which is similar to the results of Das et al. [43] Das et al. found that 50–60% of hypertensive patients do not visit health centers for their scheduled follow-up appointments, and one of the most important reasons for this was the far distance from the centers [43]. In a study investigating the determinants of the choice of health care providers in Nigeria, it was found that distance is a decisive factor in encouraging people to receive health care services [44]. The appropriate location of the centers will facilitate patients' access to these centers. Perhaps if the stage is set to teach measuring blood pressure at home, the patients’ need for going to CHCs and spending time and money due to the distance of these centers from their residence will be eliminated.

### Systemic-managerial problems

The final group of problems that lead to patients’ low utilization of CHC services are systemic-managerial problems which include: appointment of inexperienced physicians with no work experience to executive positions in the CHCs and these physicians' therapeutic vision and paying insufficient attention to disease issues. Selecting physicians with no proper executive background as health managers and not paying attention to management as a field of science will lead to creating managers without the skills and responsibilities required to have an extensive view of social issues to change people's behavior, attract participation of other organizations, and create coordination and harmony [45]. In addition, the type of management in CHCs is one of the key factors affecting productivity. According to our results, rather than being based on scientifically accepted principles, management of CHCs is more experience-driven and sometimes based on relations. As a result, such system of management cannot adapt the organization to rapid changes and provide the necessary commitment to make reforms and implement changes in the organization [46]. Under such circumstances, compulsory short-, medium-, and long-term management courses seem useful to be offered at all management levels.

Considering the major problems identified in the present study and the decrease in the access of hypertensive patients to health services due to various reasons (e.g., occurrence of crises, being busy with work and lack of sufficient time, transportation costs, distance from CHCs, and long waiting time), it seems that the self-management of high blood pressure with the supervision of CHCs is particularly important, and digital tools can play an important role in this respect. One of the most up-to-date, feasible, and accessible means is using smartphone applications [47]. This type of phones have been associated with reducing communication gap between providers and patients and may lead to improved health services. Mobile Health (mHealth) is increasingly used in medical and health fields and for self-management of various health conditions [48]. Due to the low cost of mHealth technologies, developing countries have especially used the affordances of smartphones to educate patients in order to improve service delivery [49]. In Iran, the results of a study showed that smart phones can be used as a successful tool for self-management of blood pressure in patients referring to CHCs or public hospitals [50].

According to the findings of the present study, future studies are recommended to take the following topics into consideration: (a) comparing the effectiveness of techniques such as the motivational interview as well as various measures and processes of dual process approach for attitude change in hypertensive patients to achieve better self-care; (b) investigating the effect of offering educational programs in CHCs, as an intervention, on behavior change among these patients; (c) studying the resilience of service delivery for hypertensive patients at the time of crises such as COVID-19; (d) challenges of service registration systems (such as SIB in Iran) and their effects on hypertensive patients' follow-up and utilization.

### Research limitations

This study was carried out in the CHCs of Ahvaz city, so caution must be taken when generalizing the results of this study to other settings. It should be noted that some socio-cultural factors specific to Ahvaz, including the existing multiple ethnicities, might be different in other places. In addition, some administrative aspects such as organizational climate and infrastructure may be different in Ahvaz.

## Conclusion

According to the results of this study, in order to eliminate the obstacles to hypertensive patients' utilization of CHCs’ services for the care and control of their disease, it is necessary to address two general issues of individual and systemic problems. To solve individual problems, the following solutions are proposed: 1: Giving correct and appropriate information about hypertension to raise the level of awareness of patients and their families about the disease 2: Using appropriate methods (e.g., the use of motivational interviewing techniques and the effective activity of health liaisons and volunteers in CHCs) to change negative attitudes and misconceptions of patients and increase their commitment to continuity of care 3: Providing after-hours services 4: Promoting the supportive role of charities to increase the access of low-income patients to the services needed. The solutions suggested for addressing systemic problems are as follows: 1: Holding effective training courses for health center staff 2: Increasing the motivation of employees by creating an appropriate reward system; 3: Matching the volume of services provided with the number of health center staff; 4: Equipping CHCs with the welfare facilities needed by patients; and 5: Holding training courses for managers of CHCs to change their attitudes towards first-level services and the need to pay attention to hypertension.

## Data Availability

All data generated or analyzed during this study are included in this published article [and its Additional files].
